# A Weak Shadow of Early Life Language Processing Persists in the Right Hemisphere of the Mature Brain

**DOI:** 10.1162/nol_a_00069

**Published:** 2022-05-20

**Authors:** Kelly C. Martin, Anna Seydell-Greenwald, Madison M. Berl, William D. Gaillard, Peter E. Turkeltaub, Elissa L. Newport

**Affiliations:** Center for Brain Plasticity and Recovery, Georgetown University Medical Center, Washington, DC; MedStar National Rehabilitation Hospital, Washington, DC; Children’s National Hospital, Washington, DC

**Keywords:** language, development, pediatric stroke, fMRI, lateralization, developmental plasticity

## Abstract

Studies of language organization show a striking change in cerebral dominance for language over development: We begin life with a left hemisphere (LH) bias for language processing, which is weaker than that in adults and which can be overcome if there is a LH injury. Over development this LH bias becomes stronger and can no longer be reversed. Prior work has shown that this change results from a significant reduction in the magnitude of language activation in right hemisphere (RH) regions in adults compared to children. Here we investigate whether the *spatial distribution* of language activation, albeit weaker in magnitude, still persists in homotopic RH regions of the mature brain. Children aged 4–13 (*n* = 39) and young adults (*n* = 14) completed an auditory sentence comprehension fMRI (functional magnetic resonance imaging) task. To equate neural activity across the hemispheres, we applied fixed cutoffs for the number of active voxels that would be included in each hemisphere for each participant. To evaluate homotopicity, we generated left-right flipped versions of each activation map, calculated spatial overlap between the LH and RH activity in frontal and temporal regions, and tested for mean differences in the spatial overlap values between the age groups. We found that, in children as well as in adults, there was indeed a spatially intact shadow of language activity in the right frontal and temporal regions homotopic to the LH language regions. After a LH stroke in adulthood, recovering early-life activation in these regions might assist in enhancing recovery of language abilities.

## INTRODUCTION

In the mature human brain, the cerebral hemispheres exhibit relative biases for certain cognitive abilities. The classic example is the left hemisphere bias for language processing, which is observed in the majority of adults regardless of handedness (Broca, 1861; regarding handedness: Knecht, Deppe, et al., 2000; Knecht, Dräger, et al., 2000). However, the developmental course of this bias is not fully understood. Studies of infants who suffered a unilateral brain injury provide compelling evidence that the two cerebral hemispheres are at first equivalent (*equipotential*) in the sense that either can support language when critical regions are irreversibly damaged early in development (Lenneberg, 1967, 1969). Indeed, language can develop successfully in the right hemisphere after a left hemisphere stroke around the time of birth (Fair et al., 2010; Guzzetta et al., 2008; Ilves et al., 2014; Newport et al., 2017; Staudt et al., 2001, 2002; Stiles et al., 2012; Tillema et al., 2008) or following hemispherectomy for refractory epilepsy in the first years of life (Boatman et al., 1999; Bulteau et al., 2015; Liégeois, Connelly, et al., 2008; Liégeois, Cross, et al., 2008). In the healthy brain, however, the left and right hemispheres do not engage equally during language processing: The left hemisphere exhibits language dominance that has been measured as early as the first months of life (Peña et al., 2003). This lateralization strengthens throughout childhood until it stabilizes around the age of 10 (Berl, Mayo, et al., 2014). Taken together, it appears that we begin life with a left hemisphere bias for language processing which can be overcome if there is a left hemisphere injury, but over the course of typical cognitive and brain maturation, we develop a strongly left-lateralized language system.

In the early years of typical development, in addition to the left hemisphere bias cited above, the right hemisphere also participates in the language functions that will later become strongly left-lateralized (for example, sentence processing; Berl, Mayo, et al., 2014; Gaillard et al., 2000; Holland et al., 2007). A recent study by Olulade et al. (2020) used functional magnetic resonance imaging (fMRI) to measure language activation during sentence processing in the left and right hemispheres of typically developing 4–6-year-old children, as well as of older children and adults. All participants performed an auditory sentence comprehension task, in which a button-pushing task to spoken sentences was contrasted with a button-pushing task to the same sentences played backwards (therefore unintelligible but acoustically matched to the spoken sentences). In this study, sentence comprehension robustly activated left hemisphere frontal and temporal regions at all ages. Crucially, however, Olulade et al. also found significant clusters of activity in right hemisphere frontal and temporal regions in the individual activation maps for most of the youngest children. When they compared 4–6-year-olds, 7–9-year-olds, 10–13-year-olds, and young adults, they found a continuous pattern of decline in the number of participants showing significant clusters of language activity in right frontal and temporal regions. The right frontal cortex also exhibited an age-related decline in activation magnitude. The authors suggested that the right hemisphere frontal and temporal regions that support language after early life injury are the ones also responsive during language processing in the typically developing brain. Through an unknown mechanism of brain and cognitive maturation, the magnitude of this response declines and renders language activity scarcely detectable in the right hemisphere of adults.

The consequence of this maturational change is that when an adult suffers a stroke to left hemisphere language centers, unlike what is observed after early-life stroke, language is often permanently impaired. Even when right hemisphere areas are activated during language processing in these patients, the response tends to be weak and is at best associated with limited language recovery (Martin et al., 2022; Skipper-Kallal et al., 2017a, b). How then do right hemisphere frontal and temporal regions transition from being equipotential for language processing early in life to being unavailable for language processing in the mature brain?

The answer to this question is not only critical to understanding how language may be recovered in the right hemisphere after a stroke in adulthood, but may also illuminate the principles that govern how cognitive abilities change during typical development. One possibility is that the right hemisphere regions that are equipotential for language early in life may continue to respond during language processing in adults, but only weakly. This “weak shadow” of language activity in right hemisphere regions may reflect populations of language-responsive neurons that are either fewer in number than in similar regions of the left hemisphere, or are less specifically tuned for linguistic input. An alternative possibility is that these right hemisphere regions are recruited for other cognitive functions in the mature brain, and there is no longer a spatially intact trace of the language-responsive neuronal populations that existed early in life.

An fMRI study of healthy adults by Just et al. (1996) pointed to the existence of a “weak echo” of language activation in the right hemisphere counterparts of Broca’s and Wernicke’s areas. They observed that as sentence complexity increased, the amount of activation in Broca’s and Wernicke’s areas also increased—and in homotopic right hemisphere regions, the amount of activation (which was much lower than in the left hemisphere regions) increased as well. To our knowledge, no study since Just et al.’s (1996) has intentionally investigated whether a weak language response persists in homotopic right hemisphere regions of the healthy adult brain. This investigation is important to our understanding of language system organization, and whether homotopic right hemisphere regions may be viable under certain conditions to recover language processing after stroke in adulthood.

The primary reason why homotopic language activity in the adult brain is underreported in fMRI studies is because of the way we define activity in our analyses. Most studies using fMRI to localize the neural correlates of sentence processing report only those areas with sufficiently strong activity to survive conventional statistical thresholding (e.g., voxelwise threshold of *p* < 0.001 and cluster-defining threshold of *p* < 0.05 on supra-threshold voxels). Using these cutoffs, right hemisphere areas are rarely above threshold during sentence processing in adults, or sometimes emerge but are rarely the focal point of discussion. For example, a recent fMRI study by Quillen et al. (2021) investigated the dissociation of brain region recruitment for linguistic and nonlinguistic tasks in a sample of healthy adults. Although they were not specifically examining the response in homotopic regions, they found the same pattern of activity modulation in mirroring left and right inferior frontal gyrus, pars triangularis, and posterior superior temporal sulcus for easy and difficult semantic judgments, and the same pattern of deactivation to perceptual judgments, but with a much weaker percentage signal change in the right hemisphere regions. Quillen et al. were positioned to observe this similarity because in their region of interest (ROI) selection using language activation data from an orthogonal task, they opted to include right hemisphere regions that “did not reach statistical significance but were clearly homotopic to left hemisphere language areas” (Quillen et al., 2021, p. 7). These findings accord with the observation of Just et al. (1996) that right hemisphere regions may respond similarly to core left hemisphere language regions when processing linguistic information. However, customary analysis decisions regarding what levels of activity magnitude are reported can mean that there is a relative absence of these findings in the published literature. It is possible that a weak shadow of the language engagement in right frontal and temporal regions that was observed early in life may indeed still persist in the adult right hemisphere, but we have largely been ignoring it.

One approach to defining activity that would capture the localization of both weak and strong neuronal responses is to examine a particular number of active voxels without setting a threshold for the individual *t* values. Fedorenko and colleagues routinely employ a similar method for questions about the spatial localization of language activity (Mollica et al., 2020; Shain et al., 2020). This type of *top voxel* approach would make it possible to equalize the amount of activity being assessed to determine, for example, whether the weak language response in right hemisphere regions is localized homotopic to the strong language response in left hemisphere regions. As noted above, Olulade et al. (2020) examined which brain regions were active in child and adult participants during an auditory sentence comprehension task. Their results suggest that language recruits right frontal and temporal regions with high consistency in young children but this recruitment systematically declines over age. However, a weak response to language stimuli in right frontal and temporal cortex would not survive the conventional statistical cutoff used, even at the individual level. In order to measure whether the *spatial distribution* of activity is similar on the two sides of the brain, the *quantity* of activity in the left and right hemispheres needs to be equalized.

In the current work, we wanted to determine whether language activation in homotopic right hemisphere areas persists into adulthood. We know from Olulade et al.’s (2020) findings that activation magnitude in the right hemisphere during language processing changes with age in the right hemisphere, but we do not know whether the *spatial distribution* of activation, albeit weaker in magnitude, still persists in homotopic right hemisphere regions of the mature brain. Here we investigate whether there is a weak shadow of language activity in the right frontal and temporal cortex in adults by examining whether the localization of activity is homotopic in the two hemispheres irrespective of the magnitude of this activity using a top voxel approach. We hypothesized that in adults there would indeed be a spatially intact shadow of language activation in the homotopic right frontal and temporal regions, and that the homotopicity between the left and right hemispheres in adults would be similar to the homotopicity we measured in 4–6‐, 7–9‐, and 10–13-year-old children. These results would indicate that in the mature brain, tissue that once supported language processing early in life may still be available to some degree in right hemisphere regions. This finding is potentially important for language recovery after a left hemisphere stroke in adulthood: Perhaps recovering some or all of the early-life activation in these regions might be relevant to enhancing recovery of language processing abilities in adults after stroke.

## METHODS

To investigate whether a weak shadow of language activation persists in homotopic right hemisphere regions of the mature brain, we re-analyzed the language activation data from children and adults reported in Olulade et al. (2020). Crucially, we equated the number of active voxels in the left and right hemisphere using the top voxel approach described below, and then calculated how overlapping the right hemisphere language activation was when transposed onto the activity in left hemisphere regions. This analysis decision made it possible to compare relatively weak activity in the right hemisphere to relatively strong activity in the left, to determine whether the localization of language processing is precisely homotopic at different ages. See Olulade et al. (2020) for a more comprehensive description of participants’ task and IQ (intelligence quotient) performance, MRI (magnetic resonance imaging) acquisition, and fMRI preprocessing and first-level statistical maps.

### Participants

This sample included neurologically healthy children (4–13 years old; who also served as healthy controls in Berl, Mayo, et al., 2014; Berl, Zimmaro, et al., 2014; Gaillard et al., 2007) and young adults (18–29 years old; Table 1). All included participants were right-handed, had IQs in the average or above-average range (measured using age-appropriate behavioral tests; see Olulade et al., 2020), and were native speakers of English with no significant exposure to another language before age 4.

**Table 1.** Age distribution

	Age			
	4–6-year-olds	7–9-year-olds	10–13-year-olds	Adults
*n* (females)	10 (6)	14 (8)	15 (7)	14 (7)
Age mean (*SD*)	5.5 (0.77)	8.5 (0.86)	11.4 (0.92)	21.4 (3.09)
Age range	4.5–6.7	7.4–9.8	10.0–13.0	18.4–29.1

*Note*. Adapted from Olulade et al. (2020).

### Magnetic Resonance Imaging

#### Auditory description decision fMRI task

Participants were scanned while they performed an Auditory Description Decision Task (ADDT) developed by Gaillard and colleagues (Berl, Mayo, et al., 2014; Berl, Zimmaro, et al., 2014; Gaillard et al., 2007). Participants heard short auditory descriptions of different nouns (Forward Speech condition, e.g., “a big gray animal is an elephant”) and pressed a button when they judged it to be accurate (70% of sentences were accurate descriptions), and they also heard unintelligible sound sequences (Reverse Speech condition, the same sentences from the forward condition played backwards) and pressed a button following a beep at the end (70% of sequences were followed by a beep). The 5-min task run was divided into ten 30-s blocks, each containing 10 stimuli presented every 3 s with 1 s of response time following each stimulus. The five blocks of forward and five blocks of reverse speech were interleaved, always starting with reverse speech. The frequencies of the target words in the sentences were adjusted by age group to equalize difficulty and to keep all age groups at high accuracy. Word frequencies were taken from a corpus of children’s reading materials, with the same materials used for the oldest children (10–12-year-olds) and adults (see Berl, Mayo, et al., 2014, for details). Prior to their scan, participants practiced the task on a parallel set of materials in a mock scanner to acclimate them to the environment and familiarize them with the task. All participants achieved at least 85% accuracy on the in-scanner task.

#### Scanner and auditory equipment

MRI data were collected on a 3 Tesla Siemens MAGNETOM Trio scanner with a 12-channel headcoil at Georgetown University’s Center for Functional and Molecular Imaging. Auditory stimuli for the fMRI task were presented through Sensimetrics Model S14 insert headphones, and participants wore additional Bilsom ear defenders to reduce the interference of scanner noise. Researchers confirmed that participants could clearly hear the stimuli over the scanner noise.

#### Scan sequences

A high-resolution anatomical image was collected: Siemens MPRAGE, 176 sagittal slices, TR = 1.9 s, TE = 2.52 ms, flip angle = 9 deg, 1 × 1 × 1 mm voxels, whole-brain coverage. Functional images were collected during the ADDT: echo-planar images, 50 horizontal slices, descending order, TR = 3 s, TE = 30 ms, flip angle = 90 deg, 3 × 3 × 3 mm voxels, whole-brain coverage, 100 volumes for a total of 5 min. A second run was collected if the participant moved excessively, but only one run was analyzed.

### Functional MRI Preprocessing and First-Level Statistical Maps

**Figure 1.**
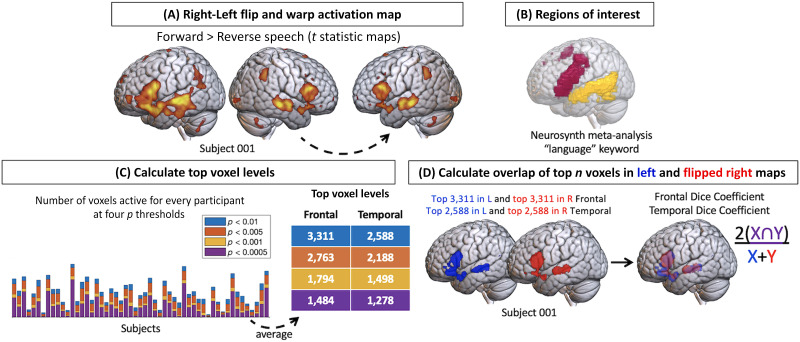
Analysis workflow. We (A) flipped the activation map for each participant, (B) masked activation in regions of interest (language regions defined by a meta-analysis database), (C) applied a top voxel cutoff to equalize the quantity of activation within the ROI in the left and flipped right maps, and (D) calculated the spatial overlap in the left and flipped right activation areas with a dice coefficient. L: left, R: right.

The first-level statistical maps from Olulade et al. (2020) were used in the current analyses. See Olulade et al. (2020) for a complete description of preprocessing and statistical analysis of these data. Briefly, functional images were slice-time corrected, realigned to the first volume of the run, co-registered to the native-space anatomical image, spatially normalized into MNI (Montreal Neurological Institute) standard space, and then smoothed (8 mm FWHM (full-width at half-maximum) Gaussian kernel) using SPM-8 (Wellcome Trust Centre for Neuroimaging at University College London, https://www.fil.ion.ucl.ac.uk/spm/doc/) and the VBM-8 toolbox (developed by Christian Gaser, University of Jena, dbm.neuro.uni-jena.de/vbm8/VBM8-Manual.pdf). Prior studies have demonstrated that adults and children in the age range we studied can be registered to a common anatomical template without introducing a significant age bias (Burgund et al., 2002; Ghosh et al., 2010). Volumes with extreme motion (>0.75 mm between volumes) were deweighted by a “bad scan” regressor in the statistical model; if the number of bad scans exceeded 25%, the run was not analyzed. The first-level general linear model included 14 predictors: Forward and reverse speech condition time courses convolved with a canonical hemodynamic response function, motion estimates for rotation and translation along the *x*, *y*, and *z* axes, the “bad scan” regressor, a global signal regressor, and a high-pass filter of four cosine basis functions. The resulting forward and reverse speech condition beta maps were contrasted using voxel-wise *t* tests to identify voxels that were more active during language processing. These activation maps (example in Figure 1A) were used in subsequent analyses.

### Flipped Activation Maps

To calculate how symmetrical (homotopic) task-related activity was within each individual activation map, we flipped each participant’s activation map across the midline (using SPM-12’s reorient utility) and normalized it to their MNI-space-warped anatomical image (using SPM-12’s normalization function). Thus their right hemisphere activation was warped to their left hemisphere anatomy in MNI space (Figure 1A). We then calculated the spatial overlap between the left hemisphere activation and the flipped right hemisphere activation to quantify how symmetrical (homotopic) language activity was for each participant (Figure 1D).

### Regions of Interest

To investigate homotopicity within the language network, we used the Neurosynth open-source online meta-analysis database (https://neurosynth.org/; Yarkoni et al., 2011) to generate a map of activation locations for all studies that reported the keyword “language” at least once in the abstract. Briefly, the Neurosynth platform calculates a two-way ANOVA (analysis of variance) to test for the association between the keyword “language” and voxel activation, and the resulting *F* values are then *z*-scored: Regions with positive *z* scores are more consistently reported as active in studies that used the term “language” than in studies that did not. These associations are then thresholded with a false discovery rate criterion of 0.01. We created separate binarized versions of the frontal and temporal areas from this map by assigning a 1 to all voxels with a z-score of 1 or greater (using SPM-12’s image calculator utility). These ROI masks (Figure 1B) were resliced to match the dimensions of the activation maps. We isolated the frontal and temporal activations for the original and flipped versions of each participant’s activation map by multiplying them by each of the binarized ROI masks. We chose to examine frontal and temporal language activations separately because these regions may have slightly different relationships with their homotopic counterparts; for example, it has been shown that lateralization stabilizes in temporal regions earlier than frontal regions in development (Berl, Mayo, et al., 2014).

### Equalizing the Activation Areas (Top Voxel Approach)

Asking whether the activity during language processing is homotopic in individual participants requires determining how overlapping the spatial locations of the activity in the left and right hemisphere are. It is well known that the *amount* of activity in the left hemisphere is greater than it is in the right, so we must equalize the quantity of activation in the left and right hemispheres before we can compare how much they overlap. To accomplish this, we determined four different cutoffs per ROI for the number of active voxels to be included for overlap comparison (Figure 1C and Table 2). These cutoffs were matched across participants and in the original and flipped activation maps. When a participant had fewer voxels with *t* values that met the statistical threshold than the average, we included the voxels with the next highest *t* values for the spatial overlap comparison. We examined the results using four different cutoffs to ensure that the spatial overlap we calculated would not be biased by the particular cutoff selected. To determine these cutoffs, for the frontal and temporal ROIs separately, we first tallied the number of voxels active in the original activation map (i.e., left hemisphere language centers) for each participant at four statistical thresholds (*p* < 0.01, 0.005, 0.001, 0.0005) with minimal cluster thresholding (*k* = 4). We then found the average number of voxels active within each ROI across all participants at each threshold, and selected these average values as the top voxel cutoffs (Figure 1C and Table 2). For each participant, we then ranked the voxels within each ROI from highest *t* value to lowest, and selected the number of top voxels equal to each cutoff. This approach ensures that the same number of voxels are examined in each participant to preclude developmental changes in activation magnitude from contaminating results. We applied the same cutoffs to both the original and flipped activation maps within each ROI to ensure that the same number of voxels were examined in the left and right hemispheres (Figure 1D). For the individual participant values that went into these averages, see supplementary materials Figure S3 and Table S1 (Supporting Information can be found at https://doi.org/10.1162/nol_a_00069).

**Table 2.** Number of voxels analyzed in regions of interest

	ROI Size	Level 1 (*p* < 0.01)	Level 2 (*p* < 0.005)	Level 3 (*p* < 0.001)	Level 4 (*p* < 0.0005)
Frontal	12,922	3,311	2,763	1,794	1,484
Temporal	12,685	2,588	2,188	1,498	1,278

### Spatial Overlap Calculation (Dice Coefficient)

We calculated a dice coefficient (Figure 1D) to summarize the number of overlapping voxels for each pair of maps (the left and right hemisphere language activations, i.e., the flipped and unflipped ADDT maps; *x* and *y*):

$$\frac{2\left(x⋂y\right)}{x+y}$$

For each participant, we averaged the dice coefficient calculated at each top voxel cutoff level to obtain one summary value of spatial overlap for each region. We grouped these values by age range to compare children to adults.

To test whether our measured dice coefficients are greater than would be measured by chance, we shuffled binary vectors the same length as the ROIs, with the same number of simulated active voxels to match each top voxel cutoff applied to the actual data, and calculated dice coefficients ten thousand times.

We also performed a between-subjects comparison of each person’s right hemisphere activity with the left hemisphere activations of all other participants in the same age group (labeled *R with Others’ L*). This comparison captures the variability in language localization in the left and right hemispheres between similarly aged participants and allows us to determine whether within-participant homotopicity exceeds the overlap arising purely from the fact that language activations are spatially similar to a degree in all subjects and all hemispheres. In this case, the *x* and *y* in the dice equation above would be one participant’s right hemisphere activation and another participant’s left hemisphere activation.

### Penetrance Maps

Penetrance maps highlight the localization of the top voxels for all participants in each age group, for the most lenient cutoff (number of top voxels corresponding to an average voxelwise *p* < 0.01 in the individual participant activation maps; Table 2 and Figure 1C). These maps, rendered on the MNI-152 standard template using MRIcroGL (https://www.nitrc.org/projects/mricrogl), visualize where language activity was the most consistent across individuals.

### Statistical Comparisons

Frequentist statistical analyses were performed using R through R-Studio (https://www.R-project.org/). Bayesian statistical analyses were performed using the JASP open-source statistical software (version 0.14.1; https://jasp-stats.org/download/). We ran separate tests for each ROI because the size of the ROIs and number of voxels compared to calculate the dice coefficients differed.

#### Homotopicity comparison (Right with Own Left)

We statistically compared the four age groups to measure whether there were age-related differences in the dice coefficients calculated for each participant’s left and right hemisphere activations (language homotopicity). We ran a one-way ANOVA for each ROI to examine the effects of age group (4–6-, 7–9-, 10–13-year-olds, and adults) on the homotopicity dice coefficient (overlap between left and flipped right activity) averaged across all four top voxel cutoff levels. Because our primary prediction was a lack of difference between age groups on the overlap comparison of right with own left (homotopicity), we also ran complementary one-way Bayesian ANOVA, which quantifies the amount of evidence in favor of the null hypothesis (no mean differences) as well as whether there is insufficient evidence to support the presence or absence of an effect.

#### Between-subjects right-left comparison (Right with Others’ Left)

We also statistically compared the language homotopicity dice coefficients to a between-subjects measure of language localization consistency in both hemispheres (a dice coefficient calculated between each participant’s right hemisphere activity and the left hemisphere activity for all other participants in the same age group). We ran a 4 × 2 mixed effects ANOVA for each ROI to examine the effects of age group (between-subjects factor) and overlap comparison (within-subjects factor with two levels: right with own left (homotopicity) and right with others’ left) on the dice coefficient measured across all four levels. We also ran a complementary 4 × 2 Bayesian repeated measures ANOVA to quantify the sufficiency of evidence in favor of the null hypothesis.

## RESULTS

**Figure 2.**
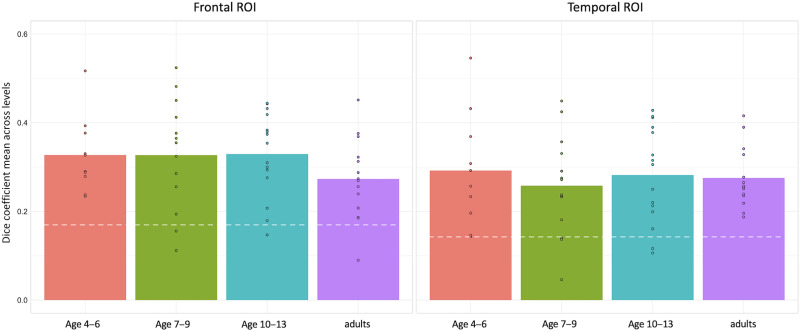
Average homotopicity for each age group. The pairwise dice coefficient averages for individual participants were binned by age group. There were no mean differences in the average homotopic overlap between age groups (means and standard deviations in Table 3, one-way ANOVAs in Tables 4 and 5). The white dashed lines indicate the dice coefficient that would be measured between random arrangements of the same number of active voxels in the same size space (0.1809 in the frontal and 0.1488 in the temporal ROI), which is substantially lower than the group means for all age groups.

When activation magnitude was equated through our top voxel method, there were no differences between the developing and mature brain in the degree to which left and right frontal and temporal activations were homotopic. The average dice coefficients (Table 3) across the four top voxel cutoffs tested (Table 2) were similar across the four age groups in the frontal and temporal ROIs (Figure 2). One-way ANOVAs measuring the effect of age group on the homotopicity dice coefficient in each ROI showed no significant main effect of age group (Table 4; Frontal: *F*(3, 49) = 1.018, *p* = 0.393; Temporal: *F*(3, 49) = 0.237, *p* = 0.87). See supplementary materials for additional correlation results (Figures S1 and S2). Under the Bayesian one-way ANOVA framework, we found that the null model predicted the observed data 3.661 times better than the age group model in the frontal ROI, and 7.813 times better in the temporal ROI, which is considered moderate evidence to support the absence of an effect of age on homotopicity (Table 5).

**Table 3.** Dice coefficient descriptive statistics

	Age			
	4–6-year-olds	7–9-year-olds	10–13-year-olds	Adults
Mean (*SD*)				
**Frontal ROI**				
R with Own L	0.33 (0.09)	0.33 (0.12)	0.33 (0.10)	0.27 (0.09)
R with Others’ L	0.23 (0.05)	0.24 (0.04)	0.21 (0.04)	0.22 (0.03)
**Temporal ROI**				
R with Own L	0.29 (0.13)	0.26 (0.11)	0.28 (0.11)	0.28 (0.07)
R with Others’ L	0.21 (0.07)	0.20 (0.09)	0.26 (0.07)	0.20 (0.04)

**Table 4.** One-way ANOVAs

Predictor	SSn	SSd	dfn	dfd	*F*	*p*
**Frontal ROI**						
Age Group	0.031	0.496	3	49	1.018	0.39
**Temporal ROI**						
Age Group	0.008	0.541	3	49	0.237	0.87

*Note*. Dice coefficient for homotopic overlap by age group. Sum of squares in the numerator (SSn) and denominator (SSd); degrees of freedom in the numerator (dfn) and denominator (dfd); *F* statistic; *p* value.

**Table 5.** Bayesian one-way ANOVAs

Models	P(M)	P(M|data)	BF_M_	error %
**Frontal ROI**				
Null model	0.500	0.785	**3.661**	
Age Group	0.500	0.215	0.273	0.003
**Temporal ROI**				
Null model	0.500	0.887	**7.813**	
Age Group	0.500	0.113	0.128	0.001

*Note*. Dice coefficient for homotopic overlap by age group. Prior model probability (P(M)); posterior model probability (P(M|data)); change from prior model odds to posterior model odds (BF_M_; here, Bayes Factor for the Null model relative to model containing Age Group, and Bayes Factor for the model containing Age Group relative to the Null model).

The dice values measuring homotopicity in our participants were substantially greater than what would be measured between random arrangements of the same number of active voxels in the same size space. The actual dice coefficient averaged across top voxel cutoff levels in the frontal ROI for adults (0.2731) was approximately 147 standard deviations above the simulated dice for the frontal ROI (average of 0.1809 and standard deviation of 0.00062775 across top voxel cutoffs); similarly, the actual temporal ROI dice for adults (0.2754) was approximately 291 standard deviations above the simulated temporal ROI dice (average of 0.1488 and standard deviation of 0.00043544 across top voxel cutoffs).

The spatial distribution of language activity is somewhat consistent across individuals (e.g., robust activation of inferior frontal and superior temporal regions), but also somewhat idiosyncratic (e.g., more variable recruitment and activation robustness of additional regions; Fedorenko & Kanwisher, 2009). Thus the high dice coefficients above might reflect general left-right symmetry of commonly activated language regions, or might additionally reflect symmetry of more idiosyncratic individualized localization of language processors. To discriminate between these two possibilities, we conducted a second reference comparison, this time comparing individual participant overlap to the overlap between each participant’s right hemisphere activation and the left hemisphere activations of the other participants in the same age group. Large overlap values between participants’ right hemisphere language activations and other participants’ left hemisphere language activations would indicate that language is localized with great consistency in the left and right hemispheres across individuals in each age group. If individuals’ own between-hemispheres overlap (homotopicity) exceeds the overlap of their right hemisphere activity with other participants’ left hemisphere activity, this would indicate that the idiosyncratic nuances of individual language networks are homotopic above and beyond the left-right symmetry of common language regions.

**Figure 3.**
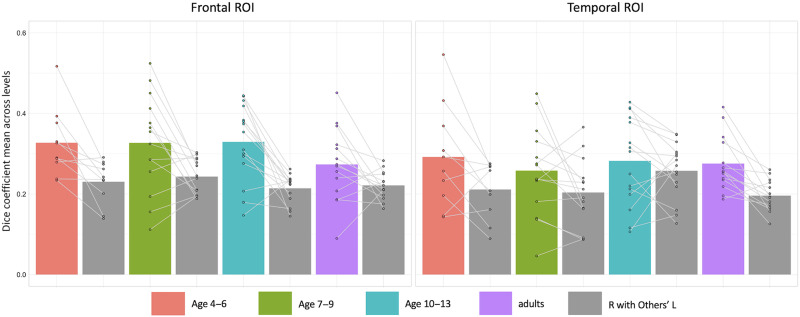
Group averages for each overlap comparison. For each participant in an age group, we compared the overlap of the activity in their right with their own left hemisphere (homotopicity) to the overlap between their right with all other participants’ left hemispheres in the age group (colorful vs. gray bars, with lines connecting individual participants) to interpret whether within-participant homotopicity was greater than between-subjects’ right-left overlap. See Table 3 for means and standard deviations, and Tables 6 and 7 for two-way mixed effects ANOVAs.

We found that within-participant homotopicity was greater than between-subjects right-left overlap in both frontal and temporal regions (Figure 3). Two-way mixed effects ANOVAs (calculated separately for the frontal and temporal ROIs) measuring the effect of age group (between-subjects factor) and overlap comparison (within-subjects factor with two levels: right with own left, and right with others’ lefts) on the dice coefficient revealed a significant main effect of overlap comparison (Frontal: *F*(1, 49) = 37.797, *p* < 0.000001; Temporal: *F*(1, 49) = 18.903, *p* < 0.0001), but no significant main effect of age group (*p* > 0.3 for both ROIs) or age group by overlap comparison interaction (*p* > 0.4 for both ROIs; Table 6). See supplementary materials for individual participant data (Figure S5). We also calculated two-way repeated measures ANOVAs using a Bayesian statistics framework. For both ROIs, the null model predicted the observed data approximately 5–8 times better than the model that included age group alone, which is considered moderate evidence to support the absence of an effect of age group on homotopicity (Table 7).

**Table 6.** Two-way mixed effects ANOVAs

Predictor	SSn	SSd	dfn	dfd	*F*	*p*	
**Frontal ROI**							
Age Group	0.022	0.325	3	49	1.128	0.347	
Overlap Comparison	**0.195**	**0.253**	**1**	**49**	**37.797**	**1.38e−07**	*
Age Group * Overlap Comparison	0.015	0.253	3	49	0.973	0.413	
**Temporal ROI**							
Age Group	0.027	0.526	3	49	0.844	0.477	
Overlap Comparison	**0.092**	**0.239**	**1**	**49**	**18.903**	**6.93e−05**	*
Age Group * Overlap Comparison	0.014	0.239	3	49	0.974	0.413	

*Note*. Dice coefficient by age group (between-subjects factor) & overlap comparison (within-subjects factor). Sum of squares in the numerator (SSn) and denominator (SSd); degrees of freedom in the numerator (dfn) and denominator (dfd); *F* statistic; *p* value.

* denotes statistically significant effects.

**Table 7.** Bayesian repeated measures ANOVA

Models	P(M)	P(M|data)	BF_M_	BF_01_	BF_10_	error %
**Frontal ROI**						
*Overlap Comparisons: R with Own L and R with Others’ L*						
Null model (includes subject)	0.200	3.235e−7	1.294e−6	1.000	1.000	
Overlap Comparison	0.200	0.775	13.790	4.174e−7	2,369,000	1.052
Age Group + Overlap Comparison	0.200	0.173	0.838	1.867e−6	5,355,301	1.010
Age Group + Overlap Comparison + Age Group * Comparison	0.200	0.052	0.218	6.273e−6	159,417	1.081
Age Group	0.200	5.364e−8	2.146e−7	**6.032**	0.166	0.649
**Temporal ROI**						
*Overlap Comparisons: R with Own L and R with Others’ L*						
Null model	0.200	0.002	0.010	1.000	1.000	
Overlap Comparison	0.200	0.759	12.569	0.003	304.903	0.743
Age Group + Overlap Comparison	0.200	0.189	0.933	0.013	75.990	1.357
Age Group + Overlap Comparison + Age Group * Comparison	0.200	0.049	0.208	0.050	19.838	1.577
Age Group	0.200	5.154e−4	0.002	**4.827**	0.207	0.389

*Note*. Dice coefficient by age group (between-subjects factor) and overlap comparison (within-subjects factor). Prior model probability (P(M)); posterior model probability (P(M|data)); change from prior model odds to posterior model odds (BF_M_); Bayes Factor for the Null model relative to current model (BF_01_); Bayes Factor for current model relative to the Null model (BF_10_).

**Figure 4.**
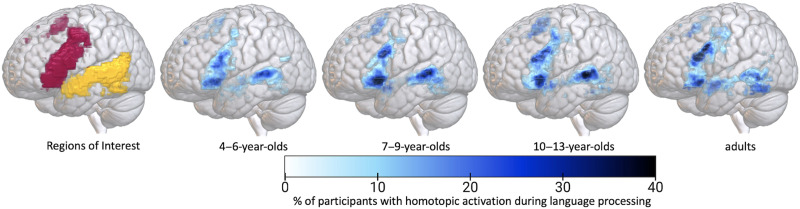
Penetrance maps showing homotopic activation consistency for each age group. Homotopic overlap maps (flipped right hemisphere activity compared to left hemisphere activity) were merged for each participant in the respective age group at the Level 1 cutoff (the top 3,311 and 2,588 voxels in the frontal and temporal regions respectively; ROI coverage displayed on the left). Darker blue areas reflect regions of greater consistency in the localization of homotopic language activity, and lighter blue areas reflect greater variance.

Penetrance maps showing the consistency of homotopic activity localization (Figure 4) reveal that the inferior frontal gyrus pars triangularis and orbitalis and mid-to-posterior superior temporal sulcus appear to be the most consistent areas of homotopic activity across age groups. Interestingly, the ventral occipitotemporal cortex, for which the left hemisphere component is implicated in reading word forms in literate adults, also appears to be homotopically activated by our auditory comprehension task with some consistency in adult but not young child participants. See supplementary materials for the penetrance maps of the activation consistency for each age group in the left and right hemisphere ROIs respectively (Figure S4).

## DISCUSSION

In the current work we investigated whether there is a weak shadow of language activity in the right frontal and temporal cortex of children and adults by using a top voxel approach to examine the degree to which the activity is similar in the two hemispheres irrespective of the magnitude of this activity. We know from prior research (Olulade et al., 2020) that activity magnitude in the right hemisphere during language processing declines with age, but we did not know whether this activity, albeit weaker in magnitude, still continues to some degree in homotopic right hemisphere regions. The current approach allowed us to investigate the location of activation while ignoring activation magnitude by comparing the same number of active voxels in the left and right hemispheres. To make this comparison, we examined dice coefficients calculated between participants’ left and flipped right hemispheres in frontal and temporal ROIs, averaged across the four top voxel cutoff levels investigated. We then grouped these values by age range to compare children to adults. We find that in older children as well as in adults there is indeed a spatially intact shadow of language activity in homotopic right frontal and temporal regions. These results indicate that in the mature brain, tissue that once supported language processing early in life may still be available to some degree in right hemisphere regions.

Twenty-five years ago, Just et al. (1996) demonstrated that as the complexity of visually presented sentences increases, the number of active voxels increases in left hemisphere Broca’s and Wernicke’s areas—as well as in their mirror-image counterparts in the right hemisphere of the healthy adult brain. The amount of activation in the right hemisphere frontal and temporal regions was much lower than in the left, but the linguistic difficulty modulated these regions in the same way. Just et al. (1996) called this observation a “weak echo” of language processing in the right hemisphere regions that are homotopic to canonical language areas. In the years since, no study to our knowledge has explicitly investigated the “weak echo” observed by Just et al. in neurotypical adults, although some have replicated it incidentally, as discussed above (e.g., Quillen et al., 2021). In the current work, we intended to bring the concept of a “weak echo,” or a “weak shadow,” to center stage. By equalizing the volume of activation in the left and right hemispheres, we find that the degree of symmetry between a person’s right hemisphere activation and their left hemisphere activation is stable from childhood through adulthood. Further, the degree of right-left symmetry is greater within individuals than the right-left symmetry across individuals of similar age, demonstrating that even idiosyncratic aspects of language organization that vary across individuals are symmetrical above and beyond typical rough symmetry that might be observed in group maps. These findings suggest that the language system is organized asymmetrically only in the sense that the left hemisphere response is stronger during language processing, but ignoring response magnitude, language activation is spatially organized in homotopic regions even in the mature brain.

### Mechanisms of Language Lateralization

In the context of evolution and ontogeny, it makes sense for language processing to recruit regions symmetrically rather than asymmetrically. Mammals are generally symmetrical across the left-to-right body axis, which is thought to be more adaptive than asymmetry: We move linearly through our environment with the support of symmetrical limbs that are controlled by the bilateral somatomotor cortices, and our bilateral sensory systems allow us to detect and react to incoming information from every angle (Concha et al., 2012; Corballis, 2009). Crucially, the brain develops along four—not three—axes: anteroposterior, dorsoventral, and then each hemisphere independently develops along its own mediolateral axis (Palmer, 2004), which generally results in symmetrical structures. The sensory and motor systems develop symmetrically (Corballis, 2009), and the language system depends on these systems for input and output. Theoretically, then, language recruitment should be somewhat symmetrical. Our results indicate that this may be the case, even in adults. Of course, other apparently lateralized systems may also be activated homotopically in the mature brain. Here we simply claim that even the language system, which is considered highly lateralized, shows substantial symmetry in the mature brain regarding which areas within the two hemispheres respond to language.

At the same time, we know that, in terms of function, there is an asymmetry in the degree to which adults rely on the hemispheres for language: Injury to left hemisphere regions, but not right hemisphere regions, in adulthood renders many central aspects of language chronically impaired. It is not well understood what properties of the left and right hemispheres create this functional asymmetry for language processing. Prominent modern theories propose that auditory neurons in the left and right hemispheres have slight biases for better processing of temporal versus spectral properties of auditory information respectively, which leads speech processing to be dominated by the left hemisphere while processing music and prosody are dominated by the right hemisphere (Albouy et al., 2020; Zatorre et al., 1992; Zatorre & Belin, 2001). Asymmetries in the size or volume of particular brain structures have also, though with notable inconsistency, been related to language lateralization, including the planum temporale (Foundas et al., 1994), Heschl’s gyrus (Penhune et al., 1996), posterior inferior frontal gyrus (Dorsaint-Pierre et al., 2006), superior longitudinal fasciculus (Powell et al., 2006), and arcuate fasciculus (Barrick et al., 2007). Sign languages in congenitally deaf individuals show the same functional asymmetries (e.g., Newman et al., 2015), so these biases must apply across modalities. All these theories focus on particular features that differ between the two hemispheres to explain not only why language lateralizes but also why it lateralizes to the left hemisphere so consistently. However, the theory that will best describe which properties of the left and right hemispheres create this functional asymmetry for language processing will need also to account for the evidence that lateralization is less strong—and is even reversible in extreme circumstances—early in life. Based on the divergence in outcomes after injury in adulthood versus early childhood, it is evident that certain properties of the neural correlates that perform language processing must change over the course of development.

One of the signs of this maturational change is a decline in the activity magnitude of homotopic right hemisphere regions during language processing. This was shown by Olulade et al. (2020) in the same sample included in the current study. It was previously unknown whether, in addition to the magnitude declining, the spatial organization of this activity also disassembles over the course of development—for example, because the right hemisphere regions that were active during language processing early in life had perhaps become dedicated to other cognitive functions. However, our results show that when one compares the same quantity of activity in the left and right hemispheres, the activation is as homotopic in adults as it is in children. We interpret this to mean that the same right hemisphere regions that are active during language processing early in life are still responsive in adults, but very weakly.

Because language can develop successfully in homotopic right hemisphere regions after early life injury to left hemisphere perisylvian cortex, it seems likely that there are language-capable processors in homotopic right hemisphere regions early in development (Newport et al., 2017, 2022). While some studies have argued that the best language outcomes after early life stroke are supported by recruitment of intact left hemisphere tissue (Raja Beharelle et al., 2010), this pattern of organization is generally observed in participants with periventricular strokes that spared left hemisphere perisylvian cortical regions, and even in these cases, the language system will sometimes recruit right perisylvian cortex (Staudt et al., 2002). Many studies, including our own (Newport et al., 2017, 2022) have found right hemisphere activation and successful language even when no left hemisphere tissue is spared. The weak activity we observe in homotopic right hemisphere areas in adults may be a weakened response of these early-life language-capable processors.

What kinds of maturational changes might lead to a weaker response to language input? The number of language-responsive neuronal populations may decline in these right hemisphere regions, for example, through the normal developmental process of synaptic pruning as a response to environmental experience. Or rather than being eliminated through pruning, it is possible that cellular inhibition of these populations may increase throughout life (e.g., through transcallosal connections or more local lateral inhibition). A third possibility is a functional rather than structural change: Language-responsive neuronal populations in these right hemisphere regions may undergo a change in their stimulus response properties, such as broadened tuning that leads to reduced specificity for the characteristics of language to which they responded earlier in life. Developmental changes in connectivity may also engender cerebral dominance for language processing. One study of two-day-old infants found strong interhemispheric connectivity that contrasted with the relatively stronger intrahemispheric connectivity observed in adults (Perani et al., 2011). In older children (age 7 and up) and adults, strong intrahemispheric (primarily left hemisphere) connectivity outweighs the strength of interhemispheric connectivity (Ailion et al., 2022; Mbwana et al., 2021). The weakening of interhemispheric connections or of intrahemispheric right hemisphere connections early in life may drive the later reduction of right hemisphere sentence processing activity over the course of childhood. Whatever the mechanisms that drive developmental reductions in right hemisphere activation, our findings constrain the possible mechanisms by demonstrating that while the level of right hemisphere activity decreases during development, the overall organization of bihemispheric regions involved in sentence processing remains symmetrical.

Rather than reflecting the presence of early-life language-capable processors, the weak language response in the right hemisphere may be a weak echo of left hemisphere language activation, that is, co-activation with the dominant left hemisphere language regions that declines in magnitude with age. This was the interpretation Just et al. (1996) made about their findings in healthy adults. Brain imaging in adults who have their hemispheres disconnected either surgically as a treatment for chronic epilepsy (*split-brain*) or by a stroke would potentially elucidate whether weak but homotopically organized right hemisphere language activation persists in the right hemisphere without transcallosal input from the left hemisphere. Unlike middle cerebral artery stroke patients, these split brain patients retain both left and right hemisphere areas for potential language processing, only without transcallosal communication. However, published fMRI studies in such patients are strikingly scarce, and studies of language fMRI tasks in these patients appear to be absent from the literature. Evidence from noninvasive brain stimulation work in healthy adults has shown that phonological decision-making is temporarily impaired by inhibiting either the left *or* right posterior inferior frontal gyrus (Hartwigsen, Price, et al., 2010), or the left *or* right supramarginal gyrus (Hartwigsen, Baumgaertner, et al., 2010). In addition, another study found that inhibiting left posterior superior temporal sulcus led to an increase in activity in the homotopic right temporal area, and improved speed in native versus foreign word recognition, though it is unclear whether the behavioral improvement was driven by the decreased activation in the left and/or the increased activation in the right temporal areas (Andoh & Paus, 2011). These findings each suggest that the language-evoked activity in right hemisphere regions reflects language processing and not merely a physiological echo of activity in the left. And, under certain conditions (e.g., suppressed left hemisphere activity), these theoretical language-capable processors in right hemisphere regions may become re-involved in language processing.

### Implications for Recovery from Aphasia

After a left hemisphere stroke in adulthood, language impairments tend to be chronic. In patients who have spared language areas in the left hemisphere, language processing continues to recruit these regions (however, see DeMarco et al., 2021), and in some cases also homotopic right hemisphere regions (Turkeltaub et al., 2011). Some studies have argued that better language outcomes after adult stroke depend on reengaging the intact left hemisphere tissue (see Anglade et al., 2014, and Turkeltaub, 2015, for a review of language recovery in the left versus right hemisphere after adult stroke). However, recovery of language functions after a left hemisphere stroke clearly relies on homotopic right hemisphere regions in some individuals: For example, several case reports have described individuals who acquired aphasia after a left hemisphere stroke, recovered substantially, and then suffered a right hemisphere stroke that caused a loss of recovered language abilities (Barlow, 1877; Basso et al., 1989; Turkeltaub et al., 2012). This right hemisphere recruitment may reflect the reengagement of the right hemisphere regions that were more strongly active during language processing early in life, a prediction that is made by Newport et al.’s Developmental Origins Hypothesis (Newport et al., 2017; see also Berl, Mayo, et al., 2014; Mbwana et al., 2009; Rosenberger et al., 2009). However, the chronic persistence of severe aphasia in most adults with large left hemisphere strokes demonstrates that the right hemisphere is often not capable of sustaining normal language ability in adults after stroke. The present results suggest that even pre-injury, these right hemisphere regions continue to be weakly active in adults during language processing. An important question raised by this finding is: If there is a weak shadow of language processing still intact in the right hemisphere, why can we not make use of it when we need to? One caveat is that in the case of stroke, the injury is so sudden and abrupt that there is no time for right hemisphere regions to transition back to processing language. In contrast, slow growing tumors in the left hemisphere do in fact produce right hemisphere language recruitment and better language outcomes compared to rapidly growing tumors (Thiel et al., 2006). If the weak shadow of early life language processing in the right hemisphere can be reanimated, it may need more time than a stroke allows.

Therapies that aim to increase right hemisphere activity in left hemisphere stroke patients to improve language outcomes have reported some success. Intention therapy, for example, trains patients to name pictures while they simultaneously perform complex movements with their left hand that activate the contralateral right hemisphere (Crosson et al., 2007; Raymer et al., 2002; Richards et al., 2002). Melodic intonation therapy trains patients to produce common words and phrases by tapping out each syllable and producing exaggerated, melodic prosody (Albert et al., 1973; Norton et al., 2009). A number of studies with small patient sample sizes have found that this combination of rhythmic motor and verbal exercises over a series of treatment sessions facilitates improvements in language production in non-fluent patients (Bonakdarpour et al., 2003; Schlaug et al., 2008; Van der Meulen et al., 2014; Wilson et al., 2006; Zumbansen et al., 2014). The benefit of these approaches may be even greater when brain stimulation is simultaneously applied: One study found that when MIT was paired with excitatory transcranial direct current stimulation to the right posterior inferior frontal gyrus for three consecutive days, stroke patients with moderate to severe non-fluent aphasia showed significant improvements in speech fluency compared to when MIT was paired with sham stimulation for the same duration in the same individuals (Vines et al., 2011). Such *rhythmic-melodic interventions* have been associated with changes in right frontal and temporal regions in adult stroke patients with aphasia (Schevenels et al., 2020; Schlaug et al., 2008; Wan et al., 2014) though studies have also reported an increase in activation of residual left hemisphere regions in some patients (Belin et al., 1996). It is possible that the spatially intact weak shadow of early life language areas in right hemisphere frontal and temporal regions may be reengaged by treatment approaches such as these in some left hemisphere adult patients with aphasia. Our between-subjects consistency results also highlight the importance of accounting for individual variability in the localization of this right hemisphere weak shadow for targeted brain stimulation treatments. Future studies should examine the presence of a weak shadow in older adults to confirm whether it is still present in this demographic, which is more similar to the age range of when adults commonly have strokes (e.g., Szaflarski et al., 2006, but without strict thresholding of activity magnitude).

### Limitations

The approach that we used to calculate how homotopic language activity was in our participants is limited to capturing active voxels that are directly parallel to one another—providing a very conservative estimate of homotopicity. Other techniques could be used to make the criterion for homotopicity more lenient, such as including right hemisphere activations that fall within some Euclidean distance from the left hemisphere language centers. Also, our participant group sizes were small (*n* = 10–14); a larger sample should be investigated to confirm the robustness of results, and with other language tasks. Finally, our interpretations about the localization of language-responsive brain regions are constrained by the univariate contrast of the two task conditions we created (activation during speech minus activation during reverse speech). Other analysis approaches that examine the patterns of response across voxels (multivariate methods) or direct measurement of the neural response using electrocorticography in special clinical populations might provide a different perspective on how the participation of right hemisphere regions in language processing changes over development.

### Conclusions

We find that a weak shadow of early life language activity persists in homotopic regions of the adult right hemisphere. These results indicate that while the strength of activity declines with age in right frontotemporal regions, weak activity during language processing is still spatially organized homotopic to core language centers. This finding is potentially important for language recovery after a left hemisphere stroke in adulthood: Perhaps recovering some or all of the early-life activation in these regions might be relevant to enhancing recovery of language abilities in adults after stroke.

## ACKNOWLEDGMENTS

This work was supported by funds from Georgetown University and MedStar Health; from the Solomon James Rodan Pediatric Stroke Research Fund, the Feldstein Veron Innovation Fund, and the Bergeron Visiting Scholars fund to the Center for Brain Plasticity and Recovery; by NIH Grants K18DC014558 to ELN, K23NS065121 to MMB, R01NS244280 to WDG, R01DC016902 to ELN and WDG, by a T32 to KM from NINDS 5T32NS041218 to Georgetown University Center for Neural Injury and Recovery; and by M01RR020359 and P30HD040677 to the IDDRC U54 HD090257 at Children’s National Health System and Georgetown University.

## FUNDING INFORMATION

Kelly C. Martin, Georgetown University (https://dx.doi.org/10.13039/100008064). Elissa L. Newport, Solomon James Rodan Pediatric Stroke Research Fund. Elissa L. Newport, Feldstein Veron Innovation Fund. Elissa L. Newport, Bergeron Visiting Scholars Fund. Elissa L. Newport, National Institutes of Health (https://dx.doi.org/10.13039/100000002), Award ID: K18DC014558. Madison M. Berl, National Institutes of Health (https://dx.doi.org/10.13039/100000002), Award ID: K23NS065121. William D. Gaillard, National Institutes of Health (https://dx.doi.org/10.13039/100000002), Award ID: R01NS244280. Elissa L. Newport, National Institutes of Health (https://dx.doi.org/10.13039/100000002), Award ID: R01DC016902. William D. Gaillard, National Institutes of Health (https://dx.doi.org/10.13039/100000002), Award ID: R01DC016902. Kelly C. Martin, National Institute of Neurological Disorders and Stroke (https://dx.doi.org/10.13039/100000065), Award ID: 5T32NS041218. The Intellectual and Developmental Disabilities Research Center U54 HD090257 at the Children’s National Health System (which also includes Georgetown University), National Institutes of Health (https://dx.doi.org/10.13039/100000002), Award ID: M01RR020359. The Intellectual and Developmental Disabilities Research Center U54 HD090257 at the Children’s National Health System (which also includes Georgetown University), National Institutes of Health (https://dx.doi.org/10.13039/100000002), Award ID: P30HD040677.

## AUTHOR CONTRIBUTIONS

**Kelly C. Martin**: Conceptualization: Supporting; Formal analysis: Lead; Funding acquisition: Supporting; Investigation: Lead; Methodology: Equal; Visualization: Lead; Writing – original draft: Lead; Writing – review & editing: Lead. **Anna Seydell-Greenwald**: Conceptualization: Supporting; Data curation: Lead; Investigation: Supporting; Methodology: Equal; Visualization: Supporting; Writing – original draft: Supporting; Writing – review & editing: Supporting. **Madison M. Berl**: Funding acquisition: Supporting; Resources: Supporting; Writing – review & editing: Supporting. **William D. Gaillard**: Funding acquisition: Supporting; Writing – review & editing: Supporting. **Peter E. Turkeltaub**: Conceptualization: Equal; Investigation: Equal; Methodology: Equal; Supervision: Equal; Visualization: Supporting; Writing – original draft: Supporting; Writing – review & editing: Supporting. **Elissa L. Newport**: Conceptualization: Equal; Funding acquisition: Supporting; Investigation: Equal; Supervision: Equal; Visualization: Supporting; Writing – original draft: Supporting; Writing – review & editing: Supporting.

## TECHNICAL TERMS

Language equipotentiality: The theory that the left and right hemispheres have equal capability to support language processing early in life.
Cerebral lateralization: A bias for one cerebral hemisphere to dominate a certain type of information processing.
Task fMRI activation magnitude: The size of the resulting *t* value at each voxel when an experimental condition is contrasted with a control condition.
Homotopic: Brain regions that are parallel to one another in opposite hemispheres.
