# Supplementary material for: A Weak Shadow of Early Life Language Processing Persists in the Right Hemisphere of the Mature Brain
Source: Neurobiol Lang (Camb). 2022 May 20;3(3):364–85. doi: 10.1162/nol_a_00069 (PMC9169899; doi:10.1162/nol_a_00069)
Supplement: Supplementary file 1 [file nol-3-3-364-s001.pdf]

Supplementary Materials for:
A weak shadow of early life language processing persists in the right hemisphere of the mature brain.
Kelly C. Martin, Anna Seydell-Greenwald, Madison M. Berl, William D. Gaillard, Peter E. Turkeltaub, Elissa L. Newport

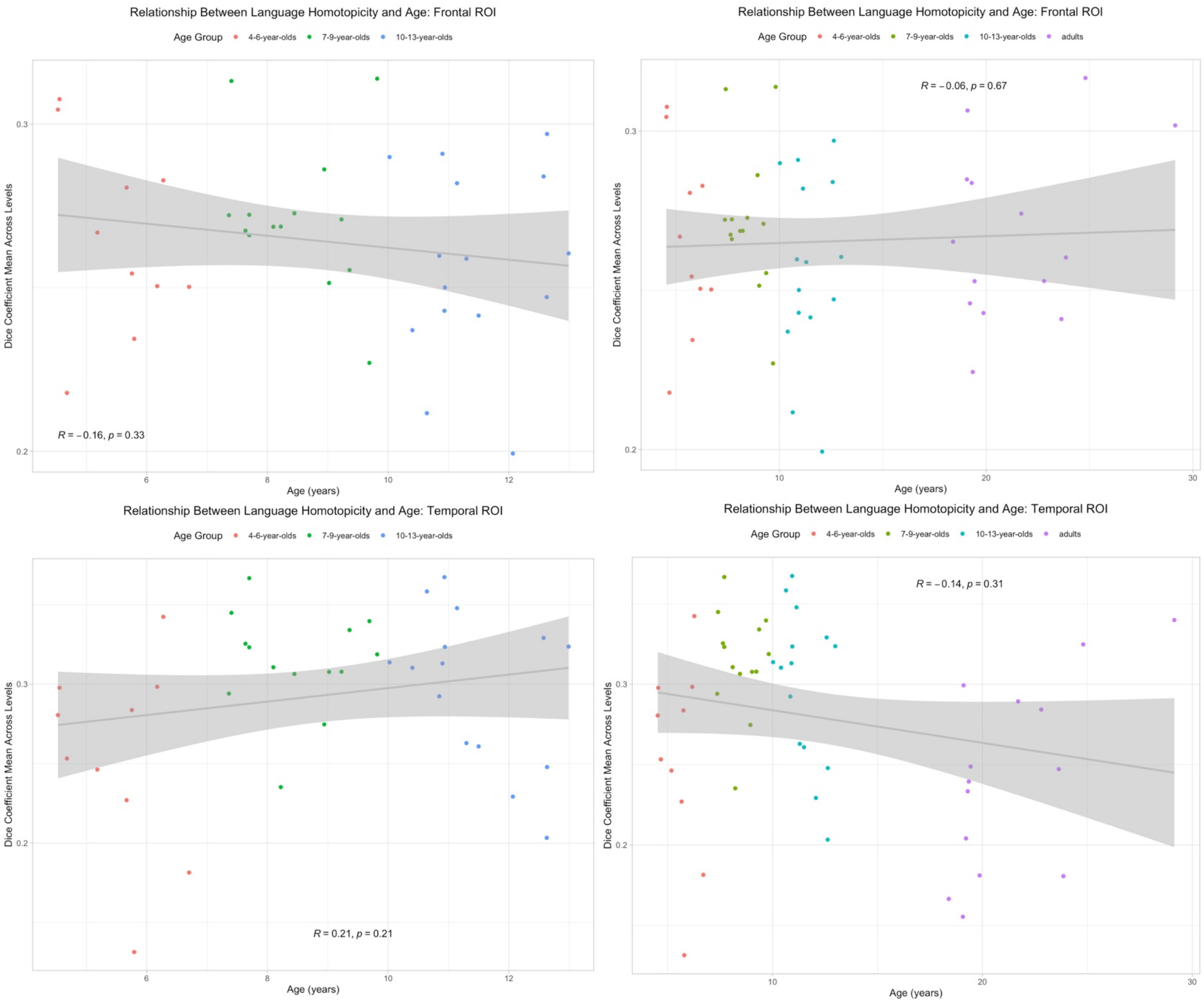

Figure S1. Homotopicity Correlations with Age (with and without Adults). The average Dice Coefficient across the four top voxel cutoffs tested, our measure of homotopic activation during language processing, was correlated with age for the three groups of children (left two plots) and for the children and adults (right two plots) in the frontal (top row) and temporal (bottom row) regions of interest. There were no significant relationships between the Dice Coefficients and age, convergent with our ANOVA results on the mean Dice Coefficients for each group (Figure 2, Tables 4 and 5).

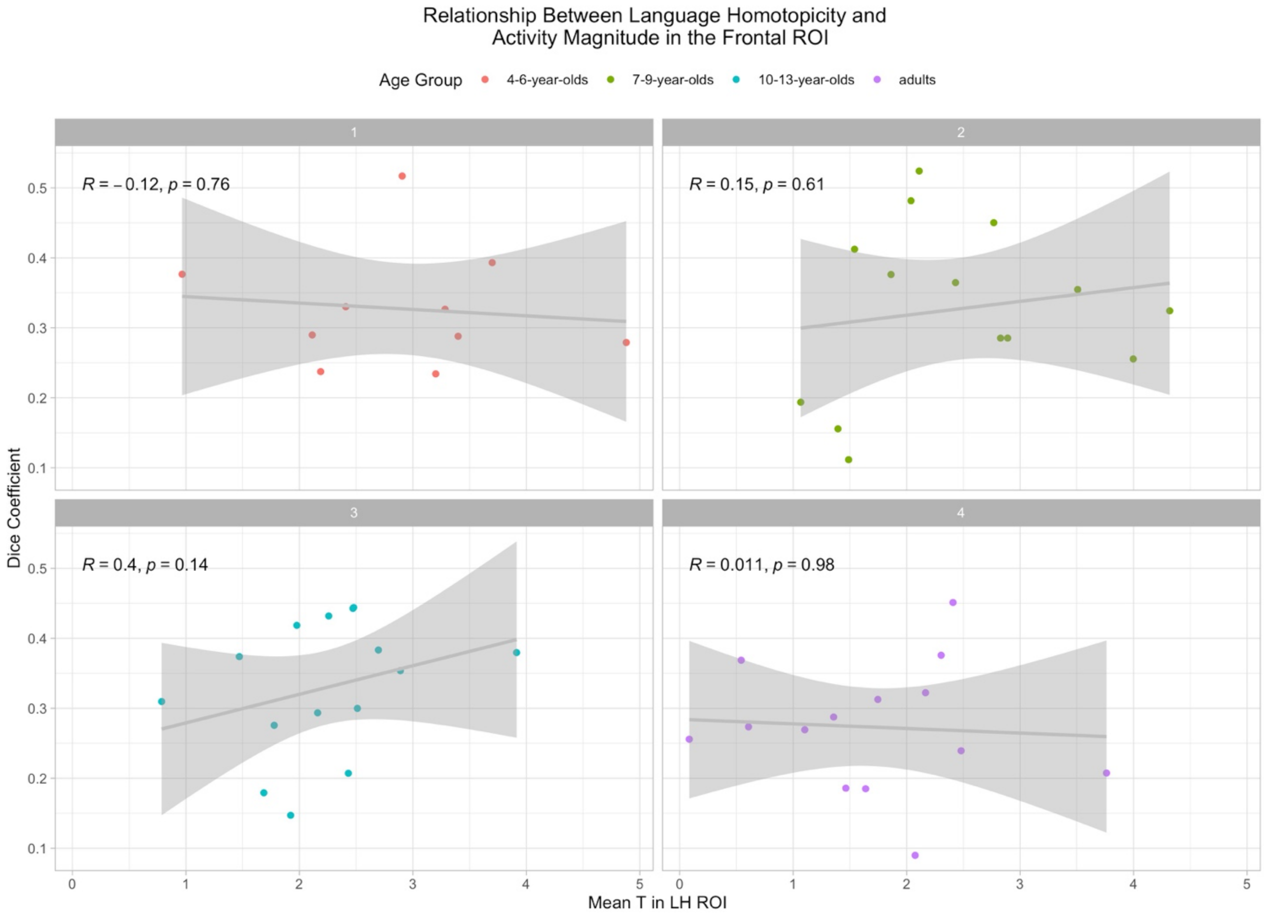
Relationship Between Language Homotopicity and
Activity Magnitude in the Frontal ROI
Age Group
4-6-year-olds
7-9-year-olds
10-13-year-olds
adults
1
2
3
4
R = −0.12, p = 0.76
R = 0.15, p = 0.61
R = 0.4, p = 0.14
R = 0.011, p = 0.98
Dice Coefficient
Mean T in LH ROI

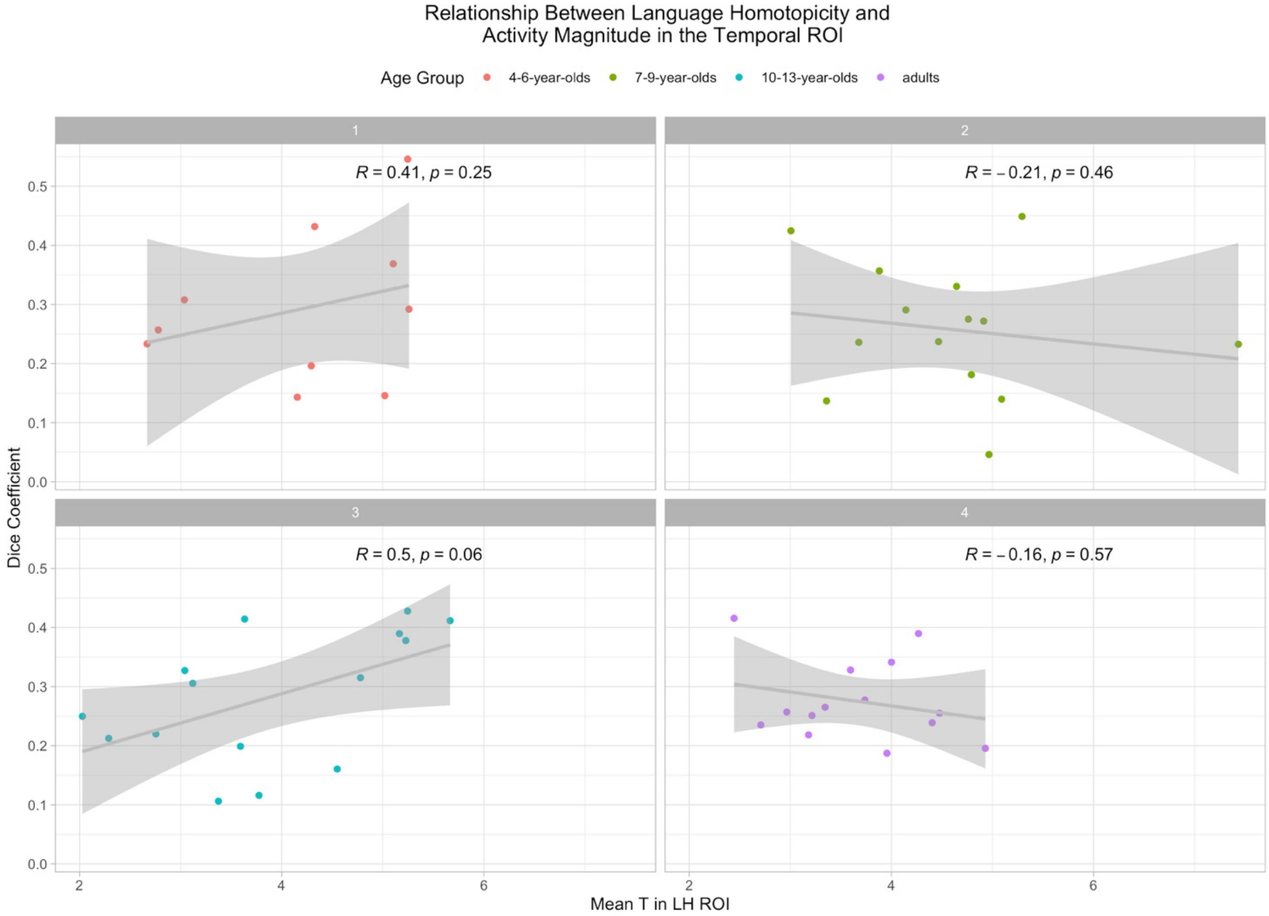
Relationship Between Language Homotopicity and
Activity Magnitude in the Temporal ROI
Age Group
4-6-year-olds
7-9-year-olds
10-13-year-olds
adults
1
2
3
4
R = 0.41, p = 0.25
R = −0.21, p = 0.46
R = 0.5, p = 0.06
R = −0.16, p = 0.57
Dice Coefficient
Mean T in LH ROI

Figure S2. Homotopicity Correlations with Mean T in LH ROI. The average Dice Coefficient across the four top voxel cutoffs tested, our measure of homotopic activation during language processing, was correlated with the magnitude of activation (mean T) in the top voxels included for the left hemisphere ROIs in the spatial overlap analysis for each age group in the frontal (top four plots) and temporal (bottom four plots) regions of interest. There were no significant relationships between the Dice Coefficients and activity magnitude in left hemisphere frontal or temporal regions, although the 10-13-year-olds had a trending positive relationship in the temporal ROI ($R=0.5$, $p=0.06$). We conclude from these results that the strength of activity in the dominant left hemisphere language regions does not drive the spatial overlap with homotopic right hemisphere regions.

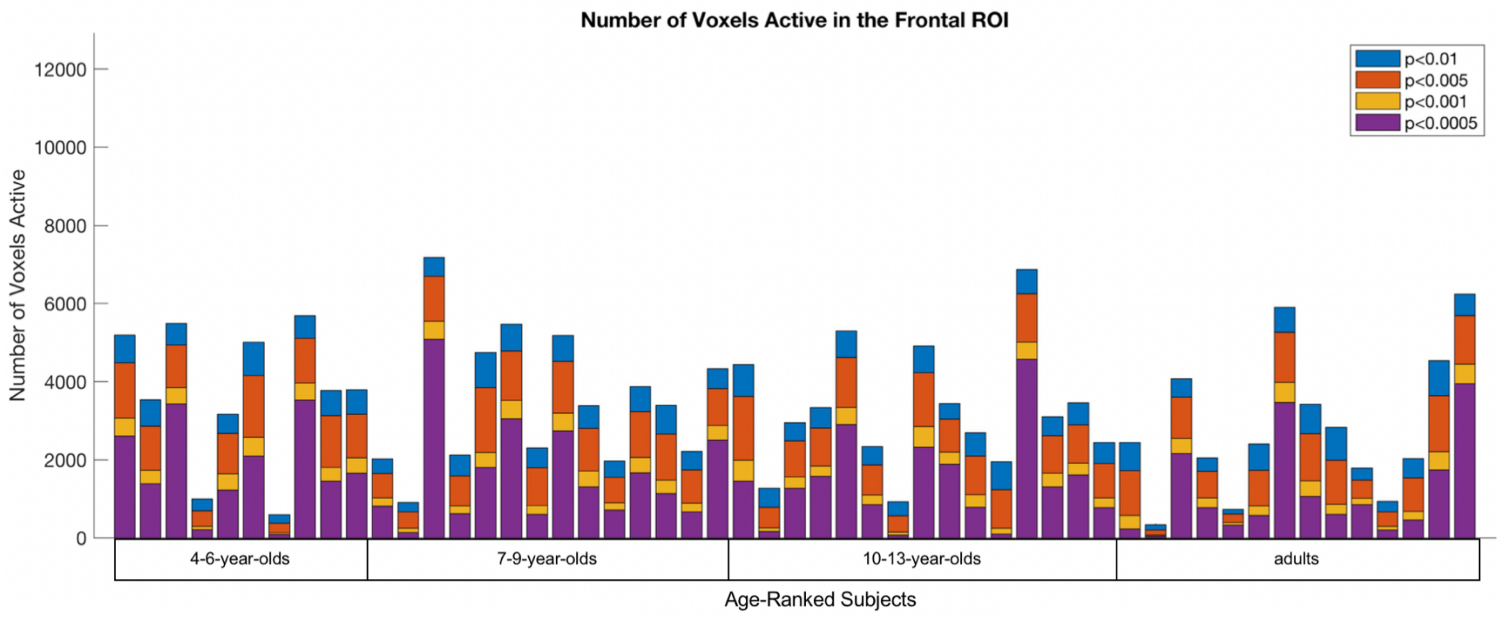

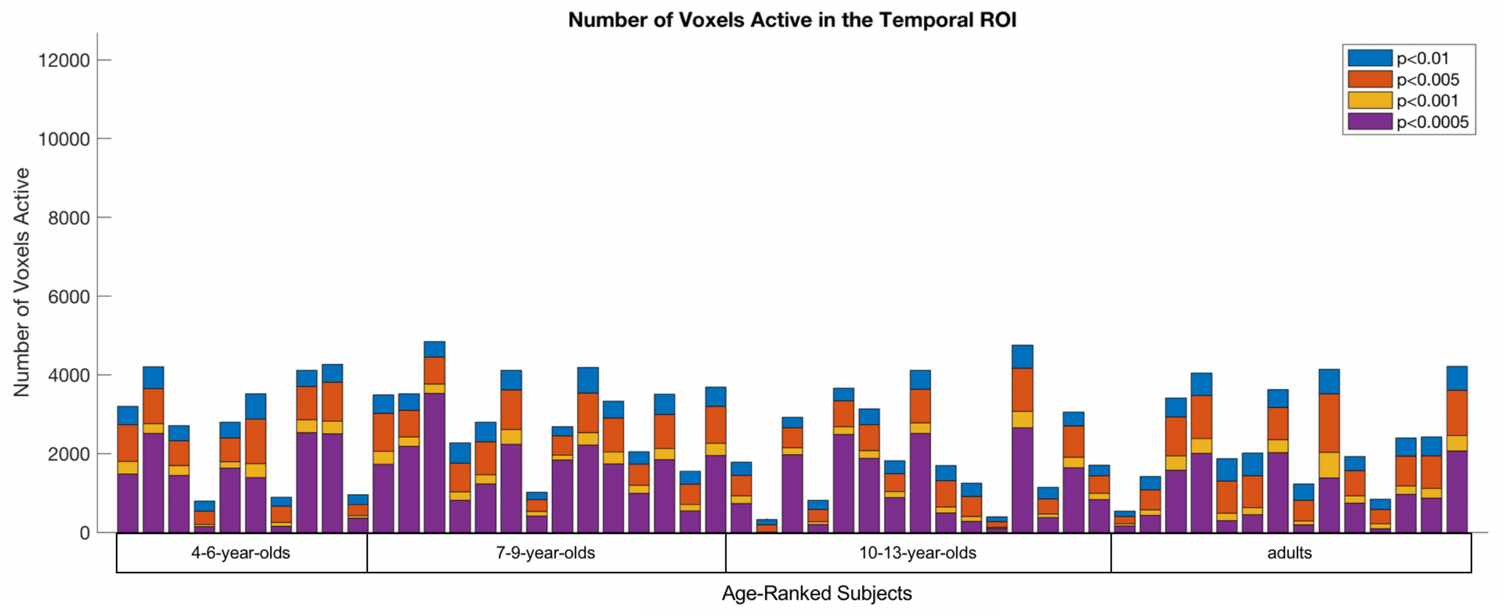

Figure S3. Number of Active Voxels at Each Cutoff. To calculate the number of top voxels to include in our spatial overlap analysis for all participants, we first thresholded each participant's Forward>Reverse Speech statistical map at four statistical cutoffs ($p<0.01$, $p<0.005$, $p<0.001$, $p<0.0005$) and counted the number of voxels that surpassed the threshold in the left hemisphere frontal and temporal ROIs respectively at each cutoff for each participant (see also Figure 1). The voxel counts for each participant at each of these statistical thresholds are visualized here, and are listed in Table S1. We then found the average number of active voxels across all participants at each threshold to determine the four top voxel cutoffs used in our spatial overlap analysis.

Frontal ROI

p<														
**4-6-year-olds**														
**p<**	**p1583**	**p130**	**p129**	**p126**	**p125**	**p180**	**p154**	**p192**	**p167**	**p117**				
**0.01**	5186	3540	5486	1002	3168	5010	591	5683	3770	3793				
**0.005**	4477	2856	4935	692	2680	4157	375	5107	3133	3168				
**0.001**	3067	1735	3848	299	1642	2576	131	3963	1811	2054				
**0.0005**	2601	1392	3427	210	1231	2100	85	3533	1453	1660				
**7-9-year-olds**														
**p<**	**p110**	**p143**	**p146**	**p1631**	**p164**	**p162**	**p124**	**p101**	**p123**	**p169**	**p136**	**p165**	**p128**	**p115**
**0.01**	2025	914	7183	2129	4745	5466	2303	5175	3387	1968	3874	3397	2216	4323
**0.005**	1658	670	6699	1589	3850	4780	1800	4520	2809	1550	3227	2656	1740	3819
**0.001**	1028	248	5543	821	2192	3519	830	3197	1714	905	2058	1480	893	2877
**0.0005**	815	137	5081	624	1807	3046	606	2744	1313	710	1676	1140	666	2502

p<															
**10-13-year-olds**															
**p<**	**p106**	**p119**	**p147**	**p108**	**p111**	**p132**	**p170**	**p113**	**p139**	**p127**	**p171**	**p105**	**p140**	**p151**	**p176**
**0.01**	4435	1275	2953	3338	5297	2339	930	4911	3435	2698	1953	6872	3101	3459	2439
**0.005**	3618	788	2484	2811	4617	1875	568	4228	3038	2098	1233	6246	2611	2897	1910
**0.001**	1989	260	1567	1848	3338	1103	152	2850	2199	1106	247	5008	1664	1918	1033
**0.0005**	1452	164	1273	1578	2902	854	67	2321	1892	780	93	4568	1311	1618	773
**Adults**															
**p<**	**a101**	**a103**	**a104**	**a105**	**a106**	**a107**	**a108**	**a109**	**a110**	**a112**	**a113**	**a114**	**a115**	**a117**	
**0.01**	2442	338	4070	2055	726	2410	5907	3417	2828	1788	934	2036	4536	6239	
**0.005**	1723	200	3598	1709	608	1732	5262	2671	1987	1485	664	1533	3636	5683	
**0.001**	578	65	2554	1026	396	819	3985	1467	866	1015	295	673	2205	4445	
**0.0005**	233	40	2160	775	329	571	3463	1064	604	860	206	457	1740	3944	

**Frontal Cutoffs**
3,311
2,763
1,794
1,484

Temporal ROI

p<														
**4-6-year-olds**														
**p<**	**p1583**	**p130**	**p129**	**p126**	**p125**	**p180**	**p154**	**p192**	**p167**	**p117**				
**0.01**	3201	4201	2710	794	2801	3514	891	4111	4269	953				
**0.005**	2733	3651	2323	539	2400	2878	662	3705	3811	707				
**0.001**	1800	2763	1692	201	1790	1752	254	2861	2821	419				
**0.0005**	1479	2514	1448	143	1629	1393	157	2534	2507	362				
**7-9-year-olds**														
**p<**	**p110**	**p143**	**p146**	**p1631**	**p164**	**p162**	**p124**	**p101**	**p123**	**p169**	**p136**	**p165**	**p128**	**p115**
**0.01**	3489	3516	4848	2270	2799	4118	1017	2683	4188	3335	2050	3506	1551	3687
**0.005**	3018	3100	4456	1754	2296	3615	830	2448	3533	2908	1728	2989	1220	3200
**0.001**	2056	2421	3769	1024	1461	2613	530	1960	2530	2043	1195	2129	707	2262
**0.0005**	1732	2188	3528	818	1232	2234	418	1833	2222	1741	991	1850	547	1952

p<															
**10-13-year-olds**															
**p<**	**p106**	**p119**	**p147**	**p108**	**p111**	**p132**	**p170**	**p113**	**p139**	**p127**	**p171**	**p105**	**p140**	**p151**	**p176**
**0.01**	1780	326	2923	810	3663	3136	1818	4116	1693	1248	392	4754	1145	3054	1701
**0.005**	1447	187	2656	582	3338	2738	1491	3634	1308	910	272	4166	851	2696	1434
**0.001**	929	7	2148	268	2684	2081	1037	2783	649	409	130	3069	466	1905	996
**0.0005**	732	0	1971	197	2483	1877	887	2509	498	276	99	2656	376	1637	830
**Adults**															
**p<**	**a101**	**a103**	**a104**	**a105**	**a106**	**a107**	**a108**	**a109**	**a110**	**a112**	**a113**	**a114**	**a115**	**a117**	
**0.01**	1701	540	1415	3415	4039	1869	2014	3626	1231	4138	1922	845	2399	2425	
**0.005**	1434	402	1080	2932	3473	1304	1433	3175	812	3517	1566	584	1932	1942	
**0.001**	996	214	577	1941	2378	481	628	2351	289	2036	930	217	1175	1113	

**Temporal Cutoffs**
2,588
2,188
1,498
1,278

Table S1. Numerical derivations of the Number of Voxels Analyzed in Regions of Interest. The voxel counts for each participant at four statistical thresholds are listed, and are also visualized in Figure S3 (see Figure 1 and Figure S3 caption for full description).

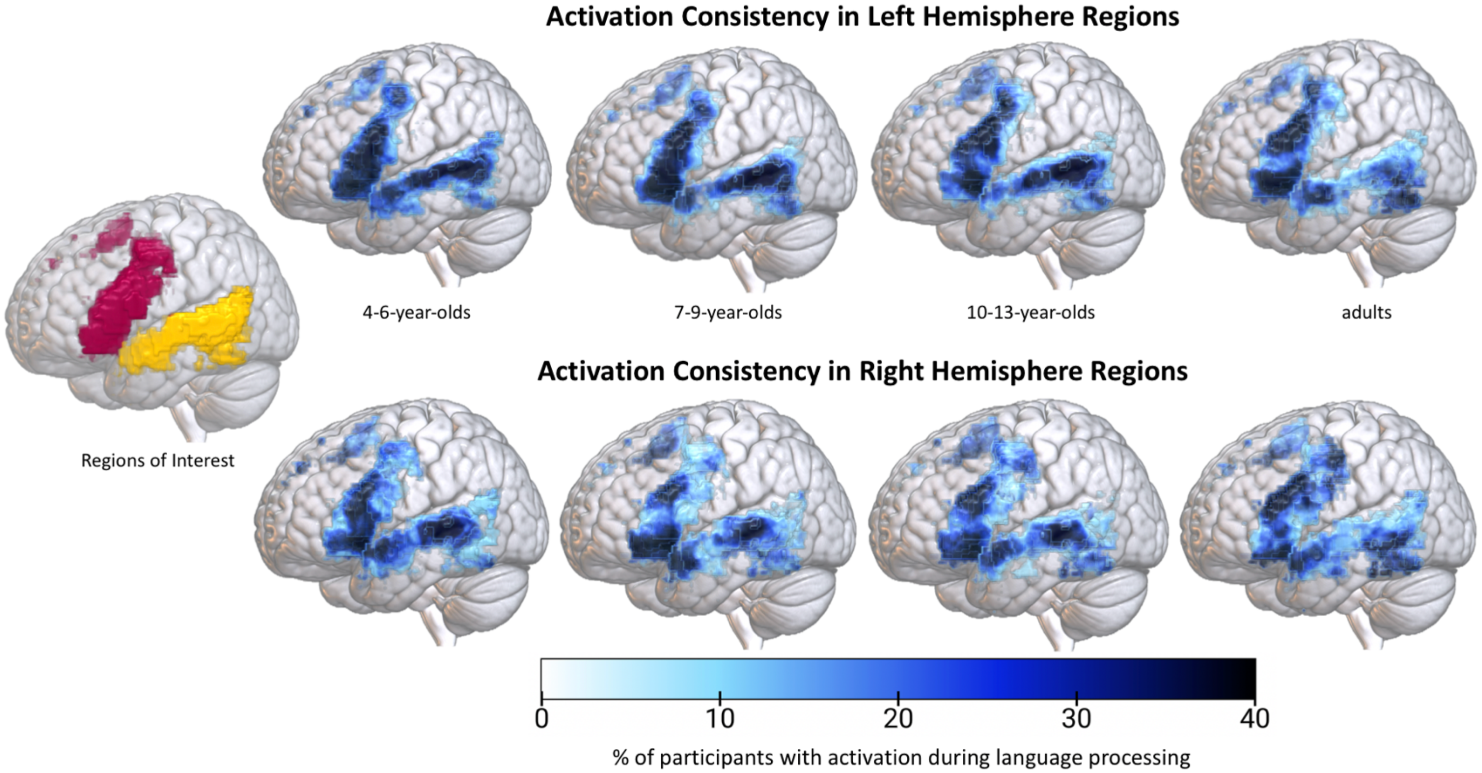

Figure S4. Penetrance Maps of Left and Right Hemisphere Consistency. The top voxel maps for the left and right hemisphere ROIs were each merged across participants in each age group, for the Level 1 top voxel cutoff (the top 3,311 and 2,588 voxels in the frontal and temporal regions respectively; ROI coverage displayed on the left). Darker blue areas reflect regions of greater consistency in the localization of language activity between participants in the age group, and lighter blue areas reflect greater variance between participants in the age group.

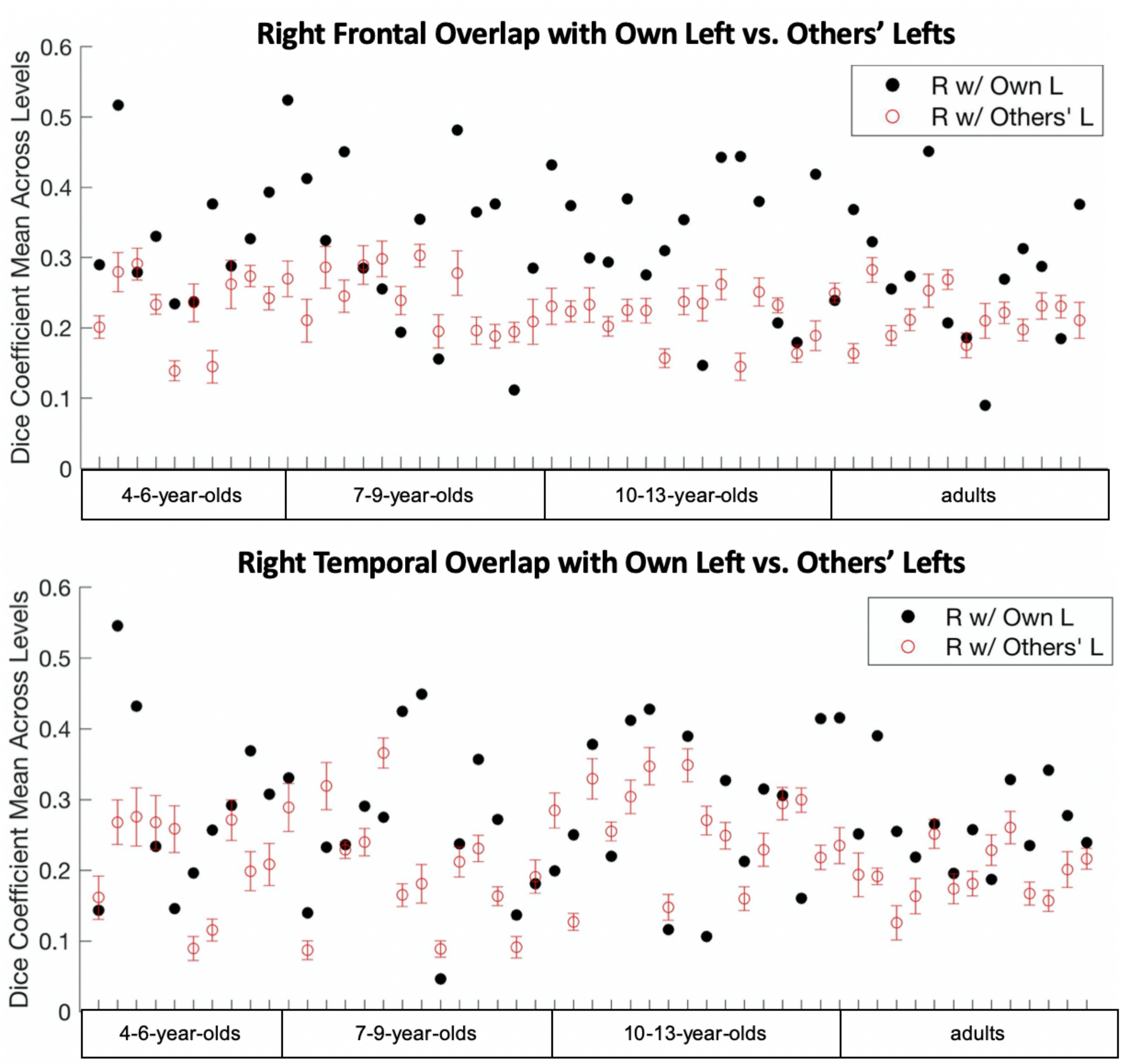

Figure S5. Individual Participant Data for Homotopicity and RH Overlap with Others' LH Activity. For each participant in an age group, we compared the overlap of the activity in their right with their own left hemisphere (homotopicity; black dots) to the overlap between their right with all other participants' left hemispheres in the age group (red circles with error bars showing standard error) to interpret whether within-participant homotopicity was greater than between-subjects right-left overlap. The group averages are reported in Figure 3 and Table 3, and statistical comparisons are reported in Tables 6 and 7. Here we show the data for the individual participants that contributed to the group averages we reported in the main paper, visualizing that the black dots representing individual homotopicity fall considerably above the red circles representing overlap with others' LHs for the majority of individuals regardless of age. There is greater consistency (qualitatively) in the between-subjects right-left overlap in the frontal ROI than in the temporal ROI; the larger number of voxels being compared for the frontal ROIs may drive this effect.
